# Comparative analysis of complete chloroplast genome of ethnodrug *Aconitum episcopale* and insight into its phylogenetic relationships

**DOI:** 10.1038/s41598-022-13524-3

**Published:** 2022-06-08

**Authors:** Conglong Xia, Manjiong Wang, Yunhui Guan, Yunfei Li, Jian Li

**Affiliations:** 1 State Key Laboratory of Bioreactor Engineering, Shanghai Key Laboratory of New Drug Design, East China University of Science and Technology, 130 Mei Long Road, Shanghai, 200237 China; 2 College of Pharmacy, Dali University, 5 Wan Hua Road, Dali, 671000 Yunnan China

**Keywords:** Molecular biology, Plant sciences, Genomic analysis, Chloroplasts, Phylogenetics

## Abstract

*Aconitum episcopale* Leveille is an important medicinal plant from the genus *Aconitum* L. of Ranunculaceae family and has been used as conventional medicine in Bai, Yi, and other ethnic groups of China. According to the available data and Ethno folk applications, *A. episcopale* is the only *Aconitum* species that has detoxifying and antialcoholic property. It can detoxify opium, especially the poisoning of *Aconitum* plants. *Aconitum* species have been widely used for their medicinal properties, and it is important to be noted that many of the species of this plant are reported to be toxic also. Distinguishing the species of this plant based on the morphology is a tough task and there are also no significant differences in the chemical composition. Therefore, before application of this plant for medicinal usage, it is very important to identify the species which could be life-threatening and exclude them. In this paper, the complete chloroplast (cp) genome sequence of *A. episcopale* was acquired by Illumina paired-end (PE) sequencing technology and compared with other species in the same family and genus. Herein, we report the complete cp genome of *A. episcopale*. The whole circular cp genome of *A. episcopale* has been found to be of 155,827 bp in size and contains a large single-copy region (LSC) of 86,452 bp, a small single-copy region (SSC) of 16,939 bp, and two inverted repeat regions (IRs) of 26,218 bp. The *A. episcopale* cp genome was found to be comprised of 132 genes, including 85 protein-coding genes (PCGs), 37 transfer RNA genes (tRNAs), eight ribosomal RNA genes (rRNAs), and two pseudogenes. A total of 20 genes contained introns, of which 14 genes contained a single intron and two genes had two introns. The chloroplast genome of *A. episcopale* contained 64 codons encoding 20 amino acids, with the number of codons encoding corresponding amino acids ranging from 22 to 1068. The Met and Trp amino acids have only one codon, and other amino acids had 2–6 codons. A total of 64 simple sequence repeats (SSRs) were identified, among which mononucleotide sequences accounted for the most. Phylogenetic analysis showed that *A. episcopale* is closely related with *A*. *delavayi*. Cumulatively the results of this study provided an essential theoretical basis for the molecular identification and phylogeny of *A*. *episcopale.*

## Introduction

**Figure 1:**
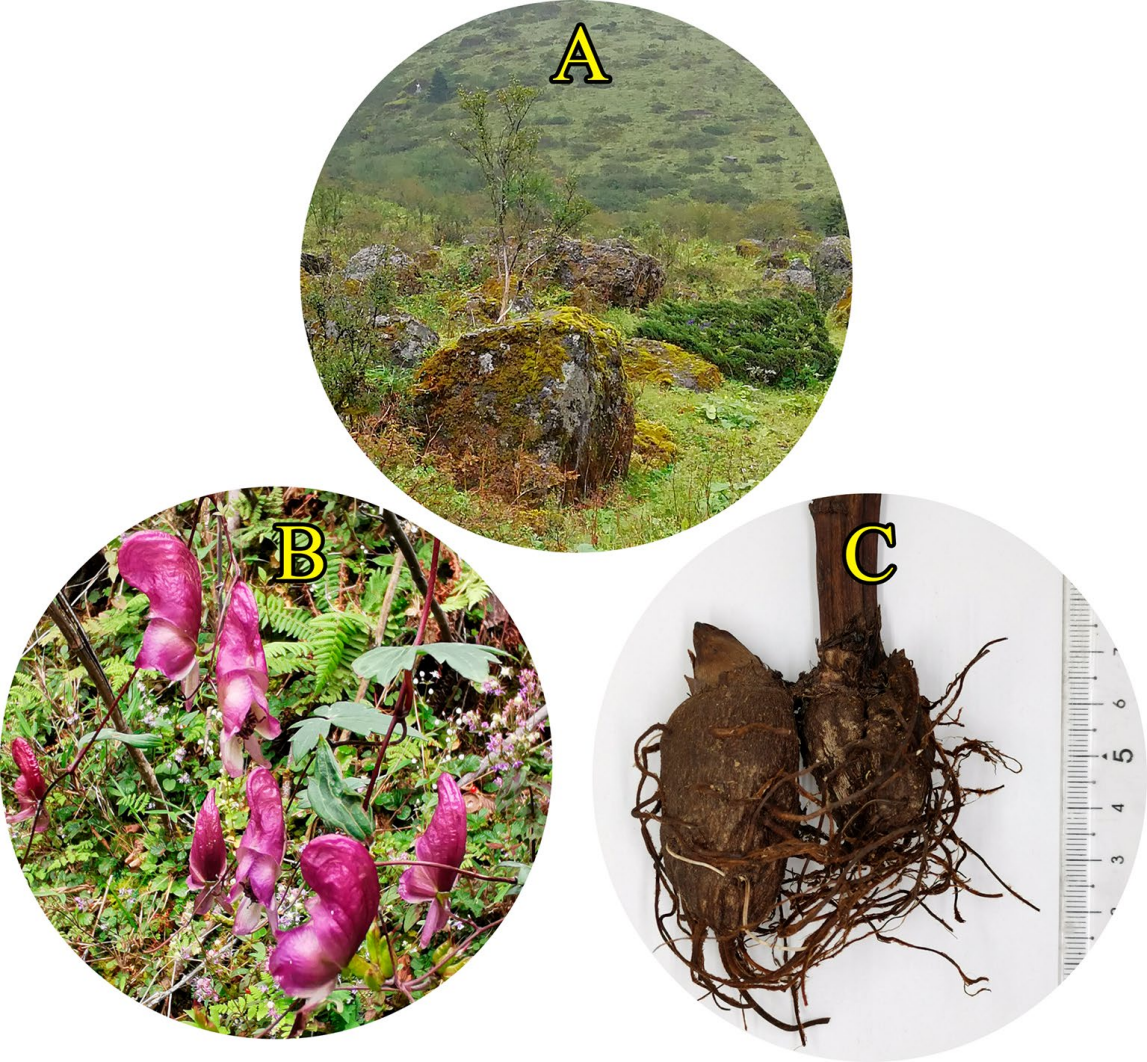
(**A**) The wild habitat of *A. ep*iscopale. (**B**) Plant of *A. episcopale*. (**C**) Crude drug from *A. episcopale*.

*Aconitum episcopale* Leveille is an important medicinal plant of the genus *Aconitum* L. of Ranunculaceae family (Fig. 1A–C), commonly known as “Hei wu tou”, “Zi wu tou”, “Dula”, “Xiao hei niu”, and so on^1^. This species is distributed mainly in the northwest Yunnan and southwest Sichuan provinces of China, where it grows in 2400–3200 m mountains and shrubs at the altitudes^2^. There are about 350 species of *Aconitum* worldwide and 170 species in China. Among these, 76 are used in folk medicine^3^, and it has been reported that most of the *Aconitum* species are highly toxic. According to the Illustrated Guide of Medicinal Plants of the Bai Nationality, *A*. *episcopale* has been found to effectively cure rheumatic bone pain, bruise injury, traumatic hematoma, joint sprain, and other diseases. Furthermore, records of Flora of China and Medicine and Pharmacy of Yi Nationality have mentioned that the *A. episcopale* can detoxify, has anti-alcoholic property, and can also detoxify opium. According to data available and ethnic application proof, *A. episcopale* Leveille is the only one species of *Aconitum* plant that can relieve the poisoning caused by the *Aconitum* plants, and the chemical component responsible for its detoxifying potential was found to be episcopalisine^4^. More than 20 compounds have been isolated from the ethanolic extract of the roots of the *A*. *episcopale*, including episcopalisine, deacetylheterophylloidine, heterophylloiding, songorine, talatisamine, 14-acetyltalaisamine, talatisamine, 14-acetyltalatisamine, vilmorrianine C, 8-deacetylyunaconitine, indaconitine, sczukininc etc. by various research groups^5–9^.

The wild population of *A*. *episcopale* has reduced significantly due to its ruthless use and habitat destruction. Many adulterates of *A. episcopale* are available in the market, which could seriously endanger people's life and health. Because the accurate distinction is difficult in the morphological appearance of these species, a molecular method is urgently needed to distinguish *A. episcopale* from the other adulterated species. Chloroplasts are independent organelles in the plant cells, have a complete set of the genome, which is relatively conserved in the genetic composition, structure and contains more abundant mutation sites. These structural features, which allow chloroplast genomes to occupy a vital position in plant species' discrimination and evolutionary study, have been widely used as super barcodes for the species identification and phylogenetic studies^10–12^. As of February 2021, more than 5000 chloroplast genomes have been recorded in the GenBank database (https://www.ncbi.nlm.nih.gov/genbank/) of the National Center for Biotechnology Information (NCBI).

For the current study, we assembled and analyzed the cp genome sequence of *A. episcopale* using Illumina PE sequencing. At the same time, the genome was compared with other published cp genomes of the same family and genus. The results determined the evolutionary position of *A. episcopale* in the Ranunculaceae family, which provided an essential theoretical basis for molecular identification and phylogeny of *A. episcopale*.

## Results

### Genome sequencing and assembly

In our study, the DNA of *A. episcopale* was paired-end sequenced using Illumina Novaseq sequencing. A total of 45,118,066 raw reads and 6,767,709,900 raw bases were gained. The proportion of Q20 (nucleotides with quality values larger than 20 in reads) was 96.54%. As a result, we obtained a scaffold with its high quality of assembled 155,827 bp in length. Finally, the entire cp genome sequence of *A. episcopale* was deposited to the GeneBank (Accession Number: MZ189733.1).

### General characteristics of chloroplast genomes from *A. episcopale*

**Figure 2:**
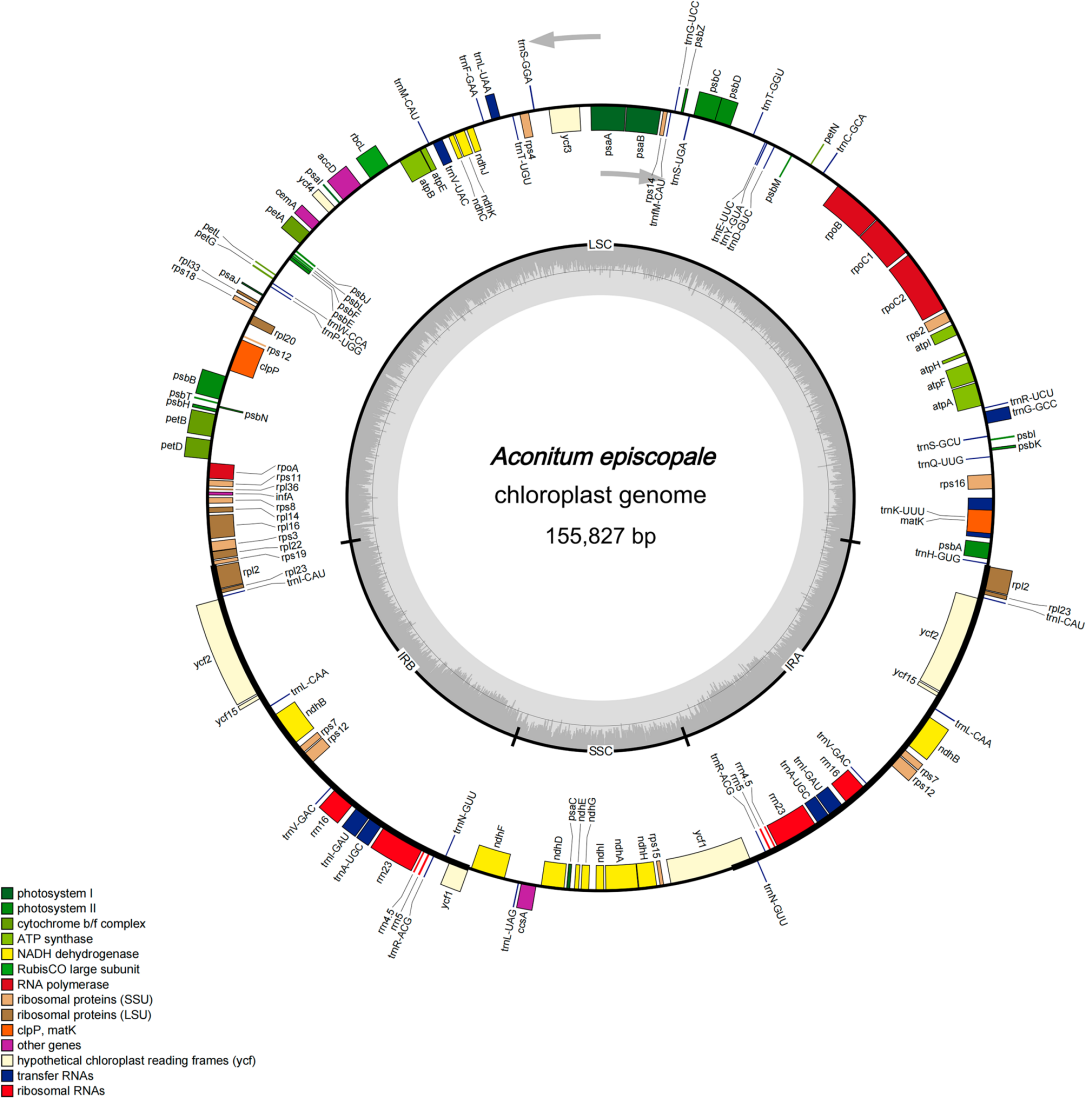
Chloroplast genome map of *A. episcopale*. Genes shown inside the circle are transcribed clockwise, and genes located outside the circle are transcribed counter-clockwise. Genes belonging to different functional groups have been colour-coded. The dashed area in the inner circle indicates the GC content of the chloroplast genome.

**Figure 3:**
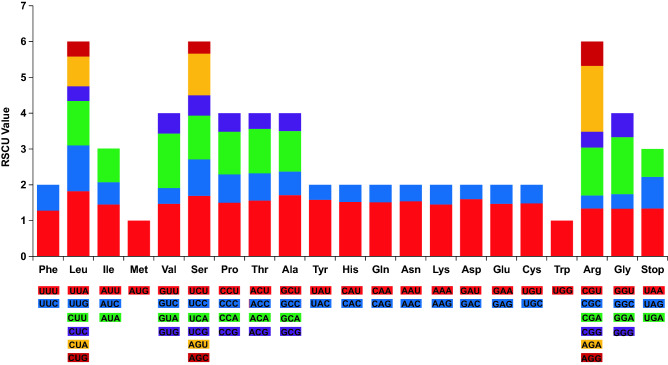
Relative synonymous codon usage (RSCU) of 20 amino acids and stop codons in all protein-coding genes of the chloroplast genome of *A. episcopale.*

The results showed that the cp genome of *A. episcopale* was 155,827 bp in length and presented a complete circular structure, including a pair of IR regions (52,436 bp), which divided the genome into an LSC region of 86,452 bp, and an SSC region of 16,939 (Fig. 2). The coding region (90,201 bp) accounted for 57.9% of the genome, and the intergenic region (65,626 bp) accounted for the remaining 42.1%. The total GC content of the cp genome was 38.09%, and the GC contents of the LSC, IR, and SSC regions were 36.2%, 43.0%, and 32.6%, respectively. Among the codons in the cp genome, the frequencies of adenine (A), thymine (T), guanine (G), and cytosine (C) were 47,849 (30.7%), 48,628 (31.2%), 29,146 (18.7%), and 30,204 (19.4%), respectively (Table 1). The *A. episcopale* chloroplast genome comprised of 132 genes, including 85 PCGs (seven duplicated genes), 37 tRNAs (seven duplicated genes), eight rRNAs (four duplicated genes), and two pseudogenes (*rps19* and *ycf1*) (Table 2). Of the 132 identified genes, 20 genes contained introns, of which 14 genes (*trnK-UUU*, *trnG-GCC*, *atpF*, *rpoC1*, *trnL-UAA*, *trnV-UAC*, *petB*, *petD*, *rpl16*, *rpl2*, *trnI-GAU*, *trnA-UGC*, *ndhA*, *ndhB*) contained one intron and other two genes (*ycf3* and *clpP)* possessed two introns. Eleven genes were situated in the LSC region, eight in the IRs region, and one in the SSC region (Table S1). Twenty genes contained Exon I, Exon II, Intron I, and their lengths were 6–775 bp, 35–1625 bp, and 493–2520 bp, respectively. The *ycf3* and *clpP* genes located in the LSC region contained Exon I, Exon II, Intron I, IntronII, and Exon III (Table S1). The protein-coding sequence of the *A*. *episcopale* cp genome contained 64 codons, and the number of codons encoding corresponding amino acids varies from 22 to 1068. Of these, 61 codons encoded 20 amino acids, and the remaining three were found to be stop codons. Methionine (Met) and tryptophan (Try) are encoded by only one codon, whereas 2–6 codons encoded the remaining amino acids (Fig. 3). Of the 64 codons encoded by the protein-coding sequence of the *A*. *episcopale* chloroplast genome, 30 had the RSCU values > 1, which were high-frequency codons. Among them, 16 codons were ending in U, 13 codons ending in A, and one codon ending in G, indicating that the third base of the high-frequency codon of *A*. *episcopale* chloroplast genome prefers to use A or U (T) (Table S2).

**Table 1:** Summary of chloroplast genome characteristics of *A. episcopale*.

Characteristics	Number
Total length (bp)	155,827
LSC length (bp)	86,452
SSC length (bp)	16,939
IRs length (bp)	52,436
Total number of genes	132
Total number of unique genes	114
Protein-coding genes (duplications)	85 (7)
tRNA gene (duplications)	37 (7)
rRNA gene (duplications)	8 (4)
Total number of pseudogenes	2
Genes duplicated in IRs	18
rRNA gene duplicated in IRs	4
Gene total length (bp)	90,201
Gene/genome (%)	57.9
Intergenetic region length (bp)	65,626
Intergenetic length/genome (%)	42.1
GC content (%)	38.09
GC content of LSC (%)	36.2
GC content of IR(%)	43.0
GC content of SSC (%)	32.6
A (bp)	47,849
T (bp)	48,628
G (bp)	29,146
C (bp)	30,204

**Table 2:** List of protein-coding genes present in the *A. episcopale* chloroplast genome.

Category	Gene group	Gene name					
Genes for photosynthesis	Subunits of photosystem I	*psaA*	*psaB*	*psaC*	*psaI*	*psaJ*	
	Subunits of photosystem II	*psbA*	*psbB*	*psbC*	*psbD*	*psbE*	*psbF*
		*psbH*	*psbI*	*psbJ*	*psbK*	*psbL*	*psbM*
		*psbN*	*psbT*	*psbZ*			
	Subunits of ATP synthase	*atpA*	*atpB*	*atpE*	*atpF*^b^	*atpH*	*atpI*
	Subunits of cytochrome	*petA*	*petB*^b^	*petD*^b^	petG	*petL*	*petN*
	Large subunit of Rubisco	*rbcL*					
	Subunits of NADH dehydrogenase	*ndhA*^b^	*ndhB*^abc^	*ndhC*	*ndhD*	*ndhE*	*ndhF*
		*ndhG*	*ndhH*	*ndhI*	*ndhJ*	*ndhK*	
Self-replication	Small subunit of ribosome	*rps2*	*rps3*	*rps4*	*rps7*^ac^	*rps8*	*rps11*
		*rps12*^ac^	*rps14*	*rps15*	*rps18*	*rps19*^a^	
	Large subunit of ribosome	*rpl2*^abc^	*rpl14*	*rpl16*^b^	*rpl20*	*rpl22*	*rpl23*^ac^
		*rpl33*	*rpl36*				
	DNA-dependent RNA polymerase	*rpoA*	*rpoB*	*rpoC1*^b^	*rpoC2*		
Other genes	Maturase	*matK*					
	Envelope membrane protein	*cemA*					
	Subunit of acetyl-CoA	*accD*					
	C-type cytochrome synthesis gene	*ccsA*					
	Protease	*clpP*^b^					
Unknown	Conserved hypothetical chloroplast reading frames	*ycf1*^ac^	*ycf2*^ac^	*ycf3*^b^	*ycf4*	*ycf15*^ac^	

^a^Genes located in the IR regions. ^b^Genes have introns. ^c^Two gene copies in IRs.

### Base composition of genome analysis

The base composition of LSC, SSC, IRS regions, and codons at different positions in the chloroplast genome of *A. episcopale* was analyzed (Table 3). In the LSC, SSC, and IRs region, AT content of SSC was the highest, followed by the LSC region and IRs region. The first, second, and third codon positions were 26,113 bp in length, and they had thymine (T) contents of 23.5–37.7%, cytosine (C) of 14.2–18.9%, adenine (A) of 28.3–31.6%, and guanine (G) of 16.5–27.0% (Table 3). The results indicated that the AT content of the chloroplast genome was higher than the GC content and also demonstrated the codon preference for using bases A and T(U).

**Table 3:** Analysis of the base composition of the genome of *A. episcopale*.

Regions	Positions	T (U) (%)	C (%)	A (%)	G (%)	Length (bp)
Total		31.2	19.4	30.7	18.7	155,827
IRa		28.7	22.3	28.3	20.7	26,218
IRb		28.3	20.7	28.7	22.3	26,218
LSC		32.4	18.5	31.4	17.7	86,452
SSC		33.3	17.4	34.1	15.2	16,939
CDS		31.1	17.9	30.5	20.5	78,339
	1st position codon	23.5	18.9	30.6	27.0	26,113
	2nd position codon	32.2	20.5	29.3	18.0	26,113
	3rd position codon	37.7	14.2	31.6	16.5	26,113

### Simple sequence repeat (SSR) analyses

**Figure 4:**
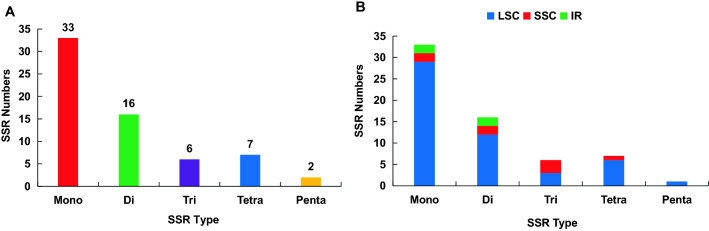
Repeat type and number analysis of SSRs in the chloroplast genome of *A. episcopale*. (**A**) Simple repeat sequence distribution in the complete cp genome. (**B**) Distribution of SSR in LSC, SSC, and IR regions.

**Figure 5:**
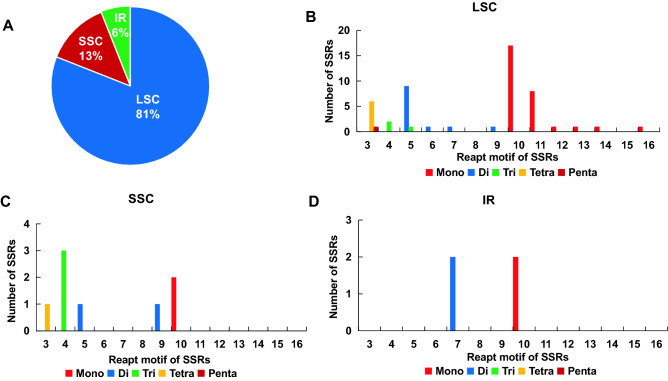
Distribution, type, and frequency of the repeat types of SSRs in the chloroplast genome of *A. episcopale*. (**A**) Presence of SSRs in the LSC, SSC, and IR regions. (**B–D**) Presence of polymers in the LSC, SSC, and IR regions.

SSRs are highly variable molecular markers within the same species and are mainly used in population genetics and polymorphism studies. These are important for gene expression, transcriptional regulation, and chromosome construction^13,14^. We investigated the type, distribution, and frequency of the repeat types of SSRs in the cp genome of *A. episcopale*. A total of 64 SSRs were identified (Fig. 4A). Among them, 52 (81%) SSRs were identified in the LSC region, including 29 mononucleotides, 12 dinucleotides, three trinucleotides, six tetranucleotides, and two pentanucleotides. The SSC region identified eight SSRs (13%), including two mono-, two di-, three tri-, and one tetra-. Four SSRs (6%) were identified in the IR regions, including two mono- and two di- (Figs. 4B, 5A).

Moreover, the different repeat motifs corresponding to the different types of SSRs were the most distributed in the LSC region, which included repeat motifs of 3, 4, 5, 6, 7, 9, 10, 11, 12, 14, and 16, while the IR regions were the least, with only two repeat motifs of 7 and 10 nucleotides. Among the five types of SSRs (mono-, di-, tri-, tetra-, penta-), the repeat motifs corresponding to mononucleotides were the most, while pentanucleotides were the least (Fig. 5B–D, Table S3).

### Long repeat analysis

**Figure 6:**
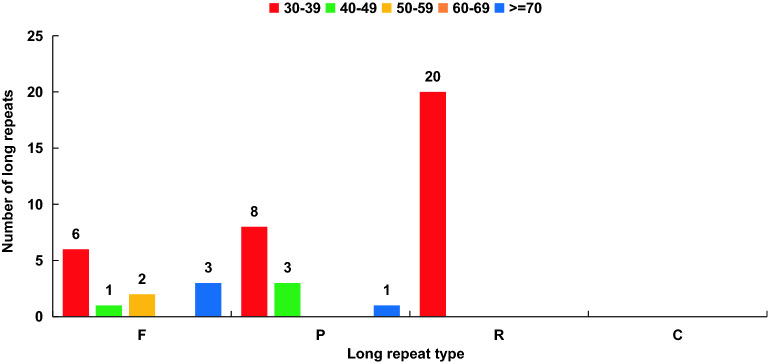
Distribution types and number of long repeats in chloroplast genomes of *A. episcopale*. REPuter was used to identify repeat sequences with length ≥ 30 bp and sequence identified ≥ 90%. F, P, R, and C indicate the repeat types F (forward), P (palindrome), R (reverse), and C (complement), respectively. Different colors indicate repeats with different lengths.

Long repeats have been found to effect the evolution, heredity, variation of life, and their indispensable role in gene expression, transcription regulation, chromosome construction, and physiological metabolism has also been established^15^. According to the structure, function, and position of the repetition, long repeats could be divided into forward repeat, reverse repeat, palindrome repeat, and complement repeat^15,16^. A total of 44 long repeats were detected in the chloroplast genome of *A. episcopale*, including 12 forward repeats (F), 20 reverse repeats (R), 12 palindromic repeats (P), and complementary repeats (C) were unfound. The length of most repeats was between 30 and 39 bp (34, 72.27%), followed by 40–49 bp (4, 9.10%), 50–59 bp (2, 4.55%), >=70 bp (3, 6.82%), and no repeats were between 60 and 69 bp. There were 11 long replicates distributed in the intergenic spacer region (non-coding region), and the remaining 33 long replicates distributed in the protein-coding region, mainly concentrated in the *rpl20*, *ycf15*, *ycf2*, and *psaB* gene regions (Fig. 6, Table S4).

### Tandem repeat analysis

Tandem repeat sequences play a significant role in the genomic rearrangement and phylogenetic analysis^17^. In this study, a total of 26 tandem repeats were detected in the *A. episcopale* cp genome, 13 tandem repeats were distributed in the gene spacers and introns (12 in the gene spacer region, one in the *clpP* intron), and the rest tandem repeats were distributed in the protein-coding region (Table S5).

### Comparison with cp genomes of other *Aconitum* species

The length of the *A. episcopale* chloroplast genome was similar to that of the *A. vilmorinianu*m (MG678799.1), *A. brachypodum* (MH886505.1), *A. pendulum* (MN7191352.1), *A. hemsleyanum* (MG678800.1), and *A. carmichaelii* (KX347251.1) chloroplast genomes (Table 4). However, the *A. episcopale* cp genome had the most extended LSC region (86,452 bp) and *A. brachypodum* cp genome had the shortest LSC region (86,292 bp). *A. episcopale*, *A. vilmorinianu*m, and *A. brachypodum* contain 132 genes, *A. pendulum*, *A. hemsleyanum* had 131 genes, and *A. carmichaelii* had only 130 genes. *A. pendulum* had 86 PCGs, 85 PCGs were present in *A. episcopale*, *A. vilmorinianu*m, *A. brachypodum,* and *A. hemsleyanum*, and 84 PCGs were in *A. carmichaelii.* Notably, all the six *Aconitum* species chloroplast genomes possessed 37 tRNA genes.

**Table 4:** Comparison of the general features of the genus *Aconitum* chloroplast genomes.

Genome feature	*A. episcopale*	*A. vilmorinianum*	*A. brachypodum*	*A. pendulum*	*A. hemsleyanum*	*A. carmichaelii*
Total length (bp)	155,827	155,761	155,651	155,662	155,684	155,737
LSC length (bp)	86,452	86,394	86,292	86,384	86,292	86,330
IR length (bp)	52,436	52,418	52,426	52,306	52,470	52,386
SSC length (bp)	16,939	16,949	16,933	16,972	16,922	17,021
Total genes	132	132	132	131	131	130
Protein gene	85	85	85	86	85	84
tRNA gene	37	37	37	37	37	37
rRNA gene	8	8	8	8	8	8
GC content (%)	38.1	38.1	38.1	38.1	38.1	38.1

### Comparative analysis of the *A. episcopale* chloroplast genome

**Figure 7:**
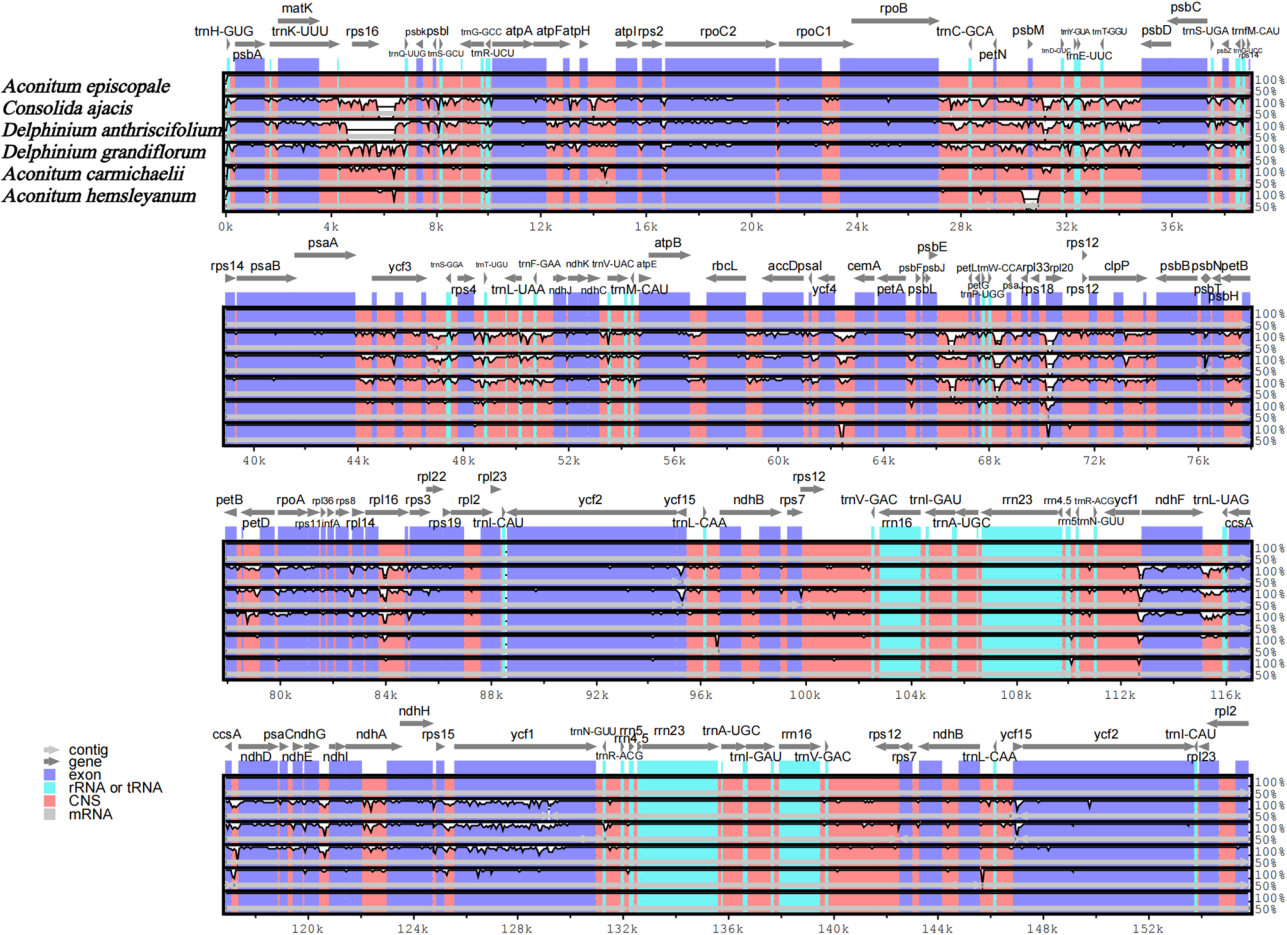
Comparison of the chloroplast genome sequences of *A. episcopale*, *A. hemsleyanum*, *A. carmichaelii*, *C. ajacis*, *D. grandiflorum*, and *D. anthriscifolium* generated with mVISTA. Grey arrows indicate the position and direction of each gene. Red and blue areas indicate the intergenic and genic regions, respectively. The vertical scale indicates the percentage of identity, ranging from 50 to 100%.

Five published sequences representing *Aconitum* (*A. hemsleyanum* and *A. carmichaelii*), *Consolida* (*C. ajacis*), *Delphinium* (*D. grandiflorum* and *D. anthriscifolium*) of the Ranunculaceae family were selected for the comparison with the sequence of *A. episcopale* to evaluate the sequence divergence of different regions of these genomes. Pairwise alignment between the chloroplast genomes of *A. episcopale* and five other species were performed using mVISTA, with the annotated *A. episcopale* chloroplast genome as a reference. We observed that the cp genome structure was conserved in the Trib. Delphineae (Fig. 7). Through pairwise comparisons of whole genomes of six species, as with the vast majority of chloroplast genomes, the IR regions were clearly more conserved than the LSC and SSC regions, with the intergenic spacer region showing the greatest variation. The most variable gene regions of the six cp genomes identified through global alignment were *rpl20*, *aacD*, *ndhF*, *ycf1*, *ycf2*, *ccsA*, and *matK*. In addition, the intergenic spacer was most variable with *matK-rps16*, *rpoB-trnC-GCA*, *petN-psbM*, *psbM-trnD-GUC*, *trnP-UGG-psaJ*, *rps18-rpl20*, *ycf3-trnS-GGA*, *ycf4-cemA*, *psbL-petL*, and *rpl16-rps3*, and the four rRNA genes (*rrn4.5*, *rrn5*, *rrn16*, and *rrn23*) were found to be most conserved.

**Figure 8:**
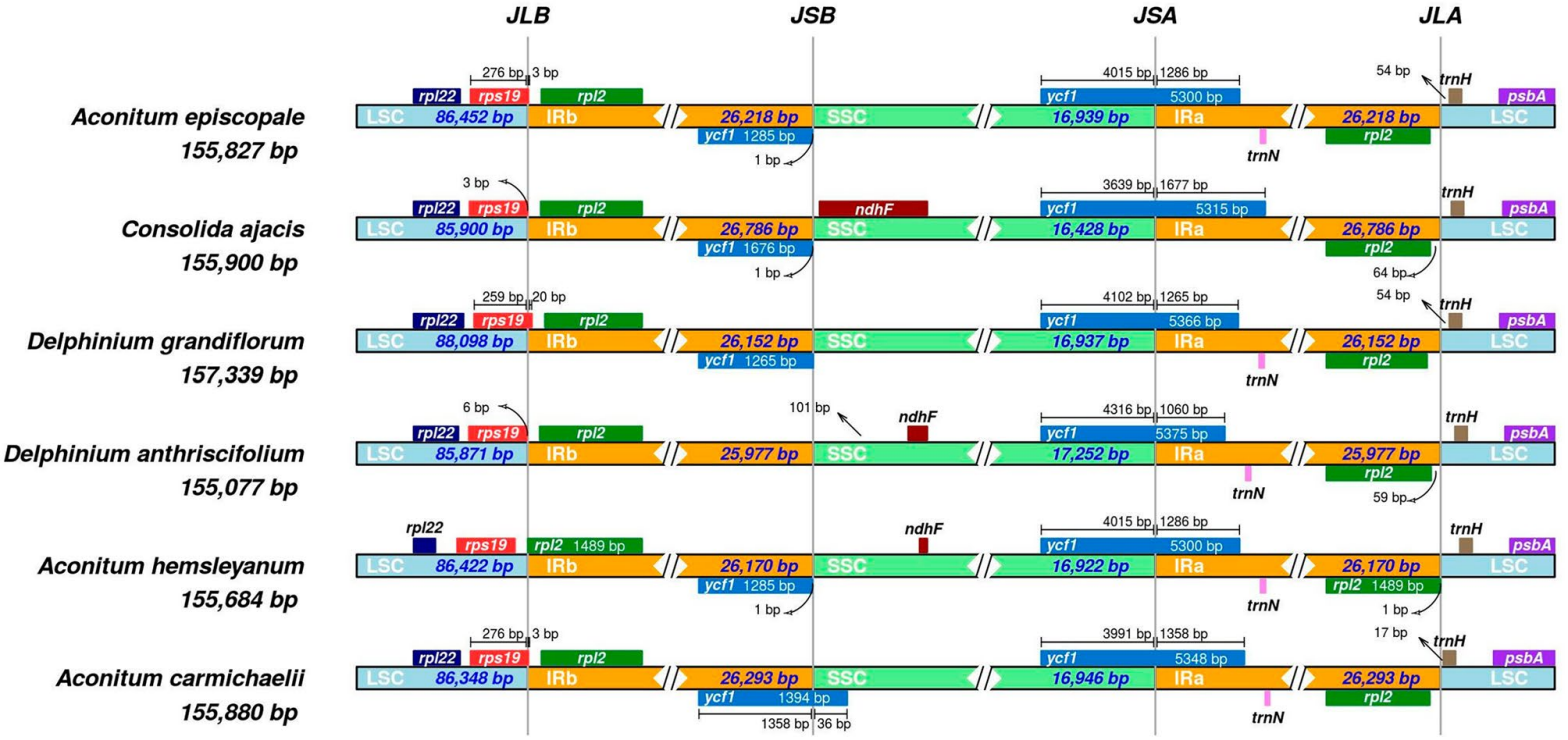
Comparison of the border regions among LSC, IR and SSC in the chloroplast genomes of *A. episcopale*, *A. hemsleyanum*, *A. carmichaelii*, *C. ajacis*, *D. grandiflorum*, and *D. anthriscifolium*. JLB: junction line between LSC and IRb; JSB: junction line between IRb and SSC; JSA: junction line between SSC and IRa; JLA: junction line between IRa and LSC.

In chloroplast genomes, IR is the most conserved region. Contraction and expansion of the IR boundary are prevalent factors affecting the chloroplast genome size^17^. The JLB boundaries of *A. episcopale*, *A. carmichaelii*, and *D. grandiflorum* were found to be located inside the *rps19* gene so that the *rps19* gene extended 3–20 bp beyond the IRb region. However, the *rps19* genes of *C. ajacis*, *D. anthraciscifolium*, *A. hemsleyanum* were at some distance from the JLB boundary. In addition, the *rps19* gene located in the JLB boundary also had a range in the length of the LSC region and IRb region, that is, between 259 and 276 bp for LSC and 3–20 bp IRb. Moreover, the JSB boundary (IRb-SSC) was found within the genes *ycf1* and *ndhF*, the region containing *ycf1* in *A. episcopale*, *A. hemsleyanum*, and *D. grandiflorum* showed some contraction at the IRb boundary, whereas the IRb region of *D. anthraciscifolium* had no *ycf1* gene. The *ndhF* genes of *A. hemsleyanum*, *C. ajacis*, and *D. anthriscifolium* showed different lengths in the SSC region, while there was no *ndhF* gene in SSC region of *A. episcopale*, *A. carmichaelii*, and *D. grandiflorum*. The JSA boundary (SSC-IRA) was located inside the *ycf1* gene in all six Trib. Delphineae species. Within the *ycf1* gene, the two parts separated by the JSA boundary (a part in the SSC region and a part in the IRa region) showed variation in length, ranging from 3639 to 4316 bp in the SSC region and 1060 bp–1677 bp in the IRa. JLA boundaries (IRa-LSC) in six species were located in the intergenic region *trnH-psbA*. The *trnH* and *pasA* also differed in length from the JLA boundary, with the *trnH* gene having the shortest length of 17 bp from the JLA boundary (Fig. 8).

### Variation zone analysis of the *A. episcopale* chloroplast genome and related species

**Figure 9:**
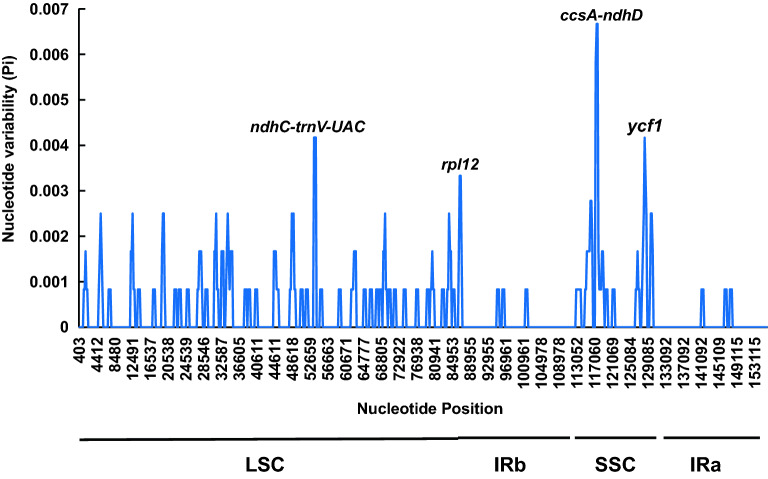
Sliding window analysis of the four *Aconitum* species chloroplast genomes.

Highly variable fragments of the chloroplast genomes can be used for phylogenetic studies and related species identification at the species level. It could also provide important information at the population level to detect the differences between the species and understand the changes in the population structure^18,19^. The cp genome sequence of *A. episcopale* was similar to that of *A. hemsleyanum*, *A. vilmorinianum*, and *A. delavayi*, which showed that the chloroplast genome of *Aconitum* was relatively conserved. Compared with the SSC region and LSC region, IR regions showed higher variability. In addition, these genomes were tested for the variation at the intervals, resulting in four highly variable regions, including two intergene regions *ndhC-trnV-UAC* (pi = 0.0042) and *ccsA-ndhD* (pi = 0.0067), two coding protein regions *rpl12* (pi = 0.0033) and *ycf1* (pi = 0.0042). These regions could further be used as highly variable fragments to identify the *Aconitum* species (Fig. 9).

### Phylogenetic analysis

**Figure 10:**
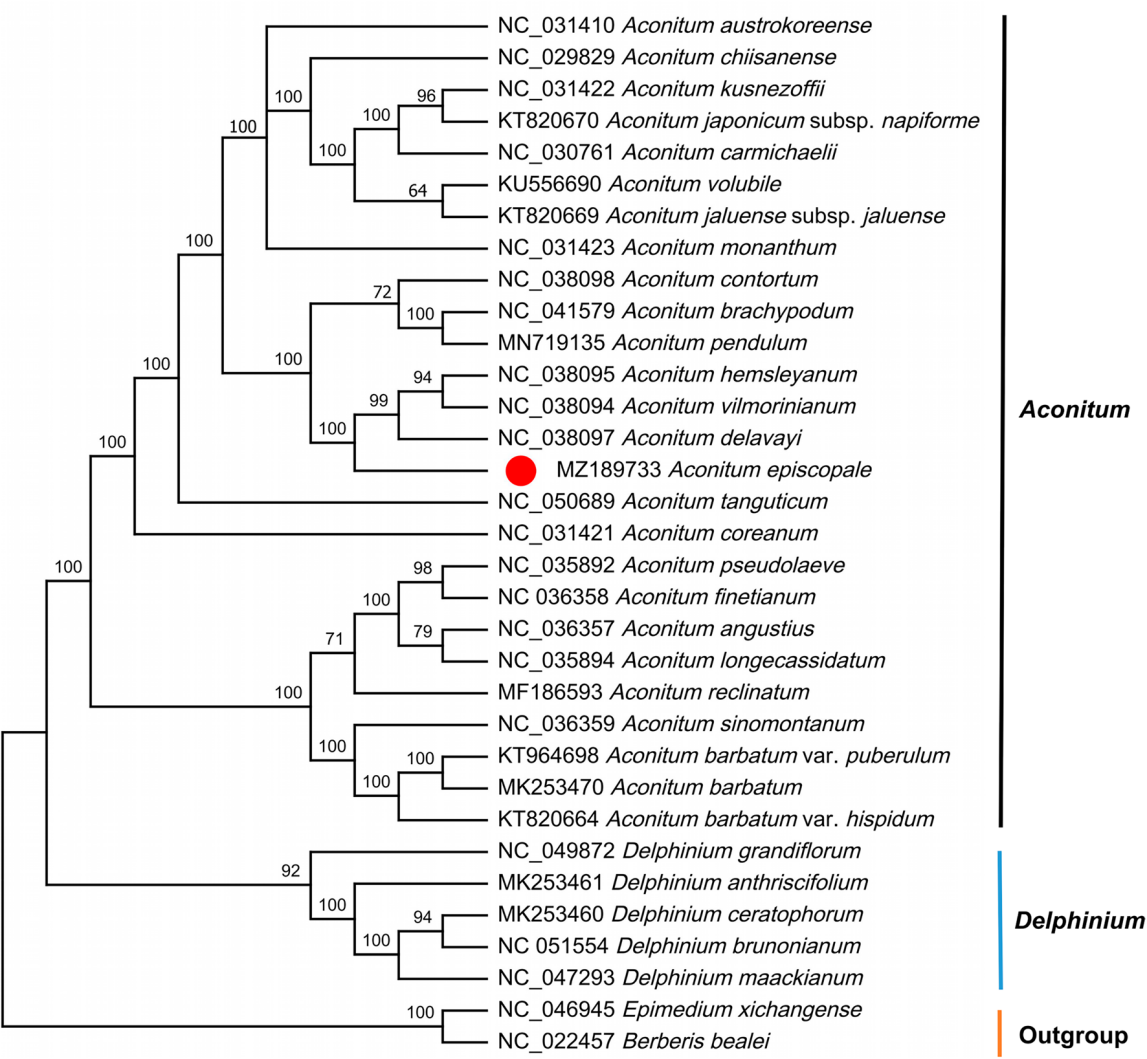
Phylogenetic tree of *A. episcopale* and 33 species of Ranunculaceae family using maximum likelihood based on the complete chloroplast genomes. Bootstrap values based on 1000 replicates have been shown on each node.

In the current study, the complete cp genome sequences of 30 species of Ranunculaceae family and that of *Berberis bealei* and *Epimedium xichangense* of Berberidaceae from the GenBank database in NCBI. In addition, the genomic sequence of *A. episcopale* was obtained by sequencing in this study. Phylogenetic trees were constructed by the maximum likelihood (ML) method to determine the relationships and phylogenetic positions of *A. episcopale* and its related species (Fig. 10). The results showed that the 33 species were divided into three large groups. The 26 species of *Aconitum* were divided into six clades. A close relationship among the *A. episcopale*, *A. delavayi*, *A. vilmorinianum*, and *A. hemsleyanum* was also uncovered.

## Discussion

In 2003, DNA barcoding was proposed by Herbert et al. as a means for species identification, which employs a short, standard stretch of DNA to enable the species identification by resolving intra- and interspecies genetic differences. Thereafter, this method has been used widely for species identification^18^. In the plant species identification, the commonly used DNA fragment contained nuclear genes ITS and ITS2, and the cp genomic sequence *rbcL*, *matK*, *psbA-trnH*^19,20^. Due to the strong identification ability of the ITS2 sequences, it has been proposed as a common DNA barcode for taxonomic identification of the medicinal plants^21^. However, common DNA barcoding does not possess sufficient variable loci for particular medicinal plant groups to identify the species such as *Fritillaria*^22^. Therefore, complete chloroplast genomes has been successfully used to identify certain medicinal plants with species closely related to it^23^. In this study, the chloroplast genome of ethnodrug *Aconitum episcopale* was sequenced in the next-generation sequencing. The chloroplast genome sequences of *Aconitum* species were found to be relatively conserved through comparative analysis, with less variation occurring between species. Through comparative analysis, we found that the differences between the chloroplast genome length, gene list, and GC contents between *A. episcopale* and other *Aconitum* species were small. In addition, we also found that the IR regions were more conserved among chloroplast genomes of *Aconitum* species than the LSC and SSC regions. This was in consistent with the results of previous comparative analytical studies of *Aconitum* species^24^. Four regions were identified as high variation regions including two intergene regions *ndhC-trnV-UAC* (pi = 0.0042) and *ccsA-ndhD* (pi = 0.0067) and two coding protein regions *rpl12* (pi = 0.0033) and *ycf1* (pi = 0.0042). These regions served as high variation segments for the species identification as described above, containing the most SNP sites, and these may serve as potential DNA barcodes for future species identification studies.

SSRs, also known as microsatellites, are highly variable molecular markers of the same species and are mainly used in the population genetics. They play an essential role in gene expression, transcription regulation, and chromosome construction. A total of 64 SSRs were detected in the cp genome of *A. episcopale*, and the results were similar to the SSR numbers detected by Kong et al. from *A*. *sinomontanum* var. *angustius* and *A*. *finetianum*^25^*.* Most mono- and dinucleotides consisted of multiple copies of A/T and AT/AT repeats, respectively. These SSRs could serve as effective biomarkers for the population genetic diversity studies of the chloroplast genome of *A. episcopale*, contributing to effective conservation measures for this medicinal plant.

## Conclusions

In the present study, we used Illumina Novaseq sequencing technology to obtain the complete chloroplast genome sequence of *A. episcopale*. Its cp genome was 155,827 bp in size and encoded 132 genes, 20 of which contained introns. The cp genome of *A. episcopale* contained 64 codons and encoded 20 amino acids, with the number of codons encoding corresponding amino acids varying from 22 to 1068. We identified a total of 64 SSRs. Four high variation regions (*ndhC-trnV-UAC*, *ccsA-ndhD*, *rpl12*, and *ycf1*) were identified by the nucleotide polymorphism analysis of the cp genome. In addition, we also performed a phylogenetic analysis of 33 whole cp genomes and colinearity analysis between *A. episcopale* and five related species. The above analytical results provided an essential theoretical basis for the molecular identification and phylogeny of the medicinal plant *A. episcopale* and valuable reference information for its effective conservation strategies.

## Materials and methods

### Sample preparation

Fresh leaves without disease spots of *A. episcopale* were collected from the Raboluo Village, Weixi County, Yunnan Province, in September 2020 (27°31′42″ N, 99°34′90″ E; elevation 2843.19 m). After that, the leaves were dried and stored with discolored silica gel. The specimen was stored in the Plant and Medicinal Herbology, College of Pharmacy, Dali University. The specimen number was recorded as WTWX001.

### Extraction of genomic DNA and sequencing

Fresh leaves of *A. episcopale* were collected, and total DNA was extracted using the E.Z.N. A® Plant DNA kit (OMEGA, Beijing). DNA quality was assessed using agarose gel electrophoresis. This DNA was further paired-end sequenced using the Illumina Novaseq 6000 (Shanghai Biozeron Biotech Co., Ltd., China) platform. Since there could have been some data with lower quality from Illumina's raw sequencing data, to make subsequent assemblies more accurate, raw data was quality clipped using Trimmomatic software (http://www.usadellab.org/cms/index.php?page=trimmomatic)^26^. The specific steps were as follows: remove the adaptor sequence in reads, remove bases containing non AGCT at the 5’ end before shearing, trim the ends of reads with sequencing quality value less than Q20, remove reads with N ratio up to 10%, discard adaptor and small fragment with length less than 75 bp after quality trimming.

### Chloroplast genome assembly, annotation, and submission

After the raw data was filtered by Trimmomatic, the NOVOPlasty (https://github.com/ndierckx/NOVOPlasty). ^27^was used to perform the chloroplast genome assembly. First, ~ 100 million reads were randomly selected and aligned to the cp genome sequence of *Aconitum delavayi* (NC_038097.1) using BWA (mem, default parameters)^28^. A perfect matched read to the *psbA* gene was selected as the seed input for NOVOPlasty. Two optional sequences, which were both single circular cp genomes, were produced, and the one with the same SSC direction to *Aconitum delavayi* was selected. Clean reads were aligned back to this sequence using BWA and inspected in IGV to exclude any assembly error. Finally, a custom-made script, which took the sequence and the corresponding bam file as input, was employed to correct ambiguous bases. The sequence was self aligned using blastn to determine the two IR regions and the start position of the LSC region. It was manually reorganized to final cp genome sequence, with the typical quadripartite structure as “LSC-IRb-SSC-IRa”. The GeSeq (https://chlorobox.mpimp-golm.mpg.de/geseq.html)^29^ was used for the gene prediction and annotation on the final assembled genome. The chloroplast genome sequences of reference used for the annotation were from *Aconitum delavayi* (NC_038097.1), *Aconitum vilmorinianum* (NC_038094.1), *Aconitum hemsleyanum* (NC_038095.1), and *Aconitum contortum* (NC_038098.1). Thereafter, the annotated sequences were submitted to GenBank. The genome was displayed using the software OrganellarGenomeDRA (http://ogdraw.mpimp-golm.mpg.de/cgi-bin/ogdraw.pl)^30^.

### Codon preference analysis

Codon usage frequency and relative synonymous codon usage (RSCU) in cpDNA from *A. episcopale* were determined using the Codon W software^31^.

### Simple repeat analysis

Simple sequence repeat, also known as microsatellites (SSRs), are widespread in the genome and generally consist of repeats of 1–6 bp to a lower extent, mainly in units of repeats of 2–3 nucleotides, such as (GA)n, (AC)n, and (GAA)n, among others. Microsatellite loci analysis was performed on the assembled *A. episcopale* chloroplast sequence genome using MISA tools^32^. The parameters were set to the definition (unit_size, min_repeats): 1–10, 2–5, 3–4, 4–3, 5–3, 6–3, and the minimum distance between two SSRs was set to 0 bp. Custom-made script is shown in "Custom-made script of MISA.txt" of supplementary file.

### Long repeat and tandem repeats analysis

Long repeat analysis was conducted using the software REPuter (http://bibiserv.techfak.uni-bielefeld.de/Repeat/)^33^ with parameters set to the minimum repeat size of 30 bp and hamming distance of three. Tandem repeats were detected using the Tandem repeats finder (https://tandem.bu.edu/trf/trf.html) online program with parameter settings chosen as default^34^.

### Comparative analysis of *A. episcopale* cp genome

The complete cp genome sequences of *Aconitum hemsleyanum* (NC_038095.1), *Aconitum carmichaelii* (KY407560.1), *Consolida ajacis* (NC_041534.1), *Delphinium grandiflorum* (NC_049872.1), and *Delphinium anthriscifolium* (MK253461.1) were from the GenBank database in NCBI (https://www.ncbi.nlm.nih.gov/genbank/). Thereafter, the comparative analysis of whole cp genome sequences of *A. episcopale* obtained by new sequencing in this study and the five published species mentioned above were carried out using the mVISTA online tool under the Shuffle-LAGAN model^35^. Genome sequence of *A. episcopale* was used as the reference sequence. The boundaries of the chloroplast genomes of the above six species were mapped using IRscope (https://irscope.shinyapps.io/irapp/)^36^. After aligning the whole chloroplast genome sequences of *A. hemsleyanum*, *A. vilmorinianum*, *A. delavayi*, and *A. episcopale*, sliding window analysis was performed by DnaSP with the parameters set to step size of 200 bp and window length of 600 bp^37^.

### Phylogenetic analysis

We obtained the cp genome sequences of 32 species from the GenBank database in NCBI (https://www.ncbi.nlm.nih.gov/genbank/) and the genome sequences of *A. episcopale* were obtained from the current research. Of these, *Berberis bealei* and *Epimedium xichangense*, two species of the Berberidaceae family, were selected as outgroups. Complete cp genome sequences of the above species and *A. episcopale* were aligned by MAFFT online tool^38^. Next, phylogenetic relationships among species were constructed using the maximum likelihood method with a bootstrap value of 1000 in IQtree software^39^. Further, images of the ML tree were processed using the software MEGA X^40^.

All the experiments were carried in accordance with national and international guidelines. The plant materials procured and used in the study comply with China’s guidelines and legislation.

## Specimen collection statement

The collection of fresh leaves obtained the permission of the owner.

## Supplementary Information

Supplementary Information 1.

Supplementary Information 2.

Supplementary Information 3.

Supplementary Information 4.

Supplementary Information 5.

Supplementary Information 6.

## Data Availability

The data supporting this study's findings are publicly available in the GenBank database of the NCBI (https://www.ncbi.nlm.nih.gov/genbank/) under accession number MZ189733.1.
