# Supplementary material for: Comparative analysis of complete chloroplast genome of ethnodrug Aconitum episcopale and insight into its phylogenetic relationships
Source: Sci Rep. 2022 Jun 8;12:9439. doi: 10.1038/s41598-022-13524-3 (PMC9178047; doi:10.1038/s41598-022-13524-3)
Supplement: Supplementary file 2 — Supplementary Information 2. [file 41598_2022_13524_MOESM2_ESM.doc]

| **Gene** | **Location** | **ExonI** | **IntronI** | **ExonII** | **IntronII** | **ExonIII** |
| --- | --- | --- | --- | --- | --- | --- |
| *trnK-UUU* | LSC | 37 | 2,520 | 35 |  |  |
| *trnG-GCC* | LSC | 23 | 720 | 47 |  |  |
| *atpF* | LSC | 145 | 734 | 410 |  |  |
| *rpoC1* | LSC | 430 | 734 | 1,625 |  |  |
| *ycf3* | LSC | 124 | 720 | 230 | 755 | 153 |
| *trnL-UAA* | LSC | 35 | 493 | 50 |  |  |
| *trnV-UAC* | LSC | 39 | 590 | 37 |  |  |
| *clpP* | LSC | 71 | 850 | 291 | 672 | 244 |
| *petB* | LSC | 6 | 798 | 642 |  |  |
| *petD* | LSC | 8 | 714 | 496 |  |  |
| *rpl16* | LSC | 9 | 1,108 | 399 |  |  |
| *rpl2* | IR | 394 | 662 | 431 |  |  |
| *ndhB* | IR | 775 | 706 | 758 |  |  |
| *trnI-GAU* | IR | 42 | 935 | 35 |  |  |
| *trnA-UGC* | IR | 38 | 800 | 35 |  |  |
| *ndhA* | SSC | 553 | 1007 | 539 |  |  |
| *trnA-UGC* | IR | 38 | 800 | 35 |  |  |
| *trnI-GAU* | IR | 42 | 935 | 35 |  |  |
| *ndhB* | IR | 775 | 706 | 758 |  |  |
| *rpl2* | IR | 394 | 662 | 431 |  |  |

**Table S1.** Locations and lengths of intron-containing genes in the *A. episcopale* chloroplast genome.
