# Supplementary material for: Comparative analysis of complete chloroplast genome of ethnodrug Aconitum episcopale and insight into its phylogenetic relationships
Source: Sci Rep. 2022 Jun 8;12:9439. doi: 10.1038/s41598-022-13524-3 (PMC9178047; doi:10.1038/s41598-022-13524-3)
Supplement: Supplementary file 3 — Supplementary Information 3. [file 41598_2022_13524_MOESM3_ESM.doc]

| **Codon** | **Number** | **Amino**  **acids** | **Ratio of Codon** | **RSCU** | **Number of**  **amino acid** | **Ratio of**  **amino**  **acid** |
| --- | --- | --- | --- | --- | --- | --- |
| UUU^*^ | 917 | Phe | 3.85% | 1.28 | 1,435 | 6.02% |
| UUC | 518 | Phe | 2.17% | 0.72 |
| UUA^*^ | 819 | Leu | 3.44% | 1.82 | 2,702 | 11.34% |
| UUG^*^ | 575 | Leu | 2.41% | 1.28 |
| CUU^*^ | 557 | Leu | 2.34% | 1.24 |
| CUC | 186 | Leu | 0.78% | 0.41 |
| CUA | 375 | Leu | 1.57% | 0.83 |
| CUG | 190 | Leu | 0.80% | 0.42 |
| AUU^*^ | 1,068 | Ile | 4.48% | 1.45 | 2,214 | 9.29% |
| AUC | 455 | Ile | 1.91% | 0.62 |
| AUA | 691 | Ile | 2.90% | 0.94 |
| AUG | 625 | Met | 2.62% | 1 | 625 | 2.62% |
| GUU^*^ | 525 | Val | 2.20% | 1.47 | 1,427 | 5.99% |
| GUC | 158 | Val | 0.66% | 0.44 |
| GUA^*^ | 541 | Val | 2.27% | 1.52 |
| GUG | 203 | Val | 0.85% | 0.57 |
| UCU^*^ | 560 | Ser | 2.35% | 1.69 | 1,990 | 8.35% |
| UCC^*^ | 337 | Ser | 1.41% | 1.02 |
| UCA^*^ | 406 | Ser | 1.70% | 1.22 |
| UCG | 188 | Ser | 0.79% | 0.57 |
| AGU^*^ | 386 | Ser | 1.62% | 1.16 |
| AGC | 113 | Ser | 0.47% | 0.34 |
| CCU^*^ | 423 | Pro | 1.78% | 1.50 | 1,129 | 4.73% |
| CCC | 222 | Pro | 0.93% | 0.79 |
| CCA^*^ | 336 | Pro | 1.41% | 1.19 |
| CCG | 148 | Pro | 0.62% | 0.52 |
| ACU | 526 | Thr | 2.21% | 1.56 | 1,350 | 5.66% |
| ACC | 255 | Thr | 1.07% | 0.76 |
| ACA^*^ | 419 | Thr | 1.76% | 1.24 |
| ACG | 150 | Thr | 0.63% | 0.44 |
| GCU^*^ | 596 | Ala | 2.50% | 1.71 | 1,393 | 5.84% |
| GCC | 229 | Ala | 0.96% | 0.66 |
| GCA^*^ | 393 | Ala | 1.65% | 1.13 |
| GCG | 175 | Ala | 0.73% | 0.5 |
| UAU^*^ | 560 | Tyr | 2.35% | 1.58 | 897 | 3.76% |
| UAC | 337 | Tyr | 1.41% | 0.42 |
| CAU^*^ | 423 | His | 1.78% | 1.52 | 645 | 2.70% |
| CAC | 222 | His | 0.93% | 0.48 |
| CAA^*^ | 336 | Gln | 1.41% | 1.51 | 484 | 2.03% |
| CAG | 148 | Gln | 0.62% | 0.49 |
| AAU^*^ | 526 | Asn | 2.21% | 1.54 | 781 | 3.27% |
| AAC | 255 | Asn | 1.07% | 0.46 |
| AAA^*^ | 419 | Lys | 1.76% | 1.45 | 569 | 2.38% |
| AAG | 150 | Lys | 0.63% | 0.55 |
| GAU^*^ | 596 | Asp | 2.50% | 1.60 | 825 | 3.46% |
| GAC | 229 | Asp | 0.96% | 0.40 |
| GAA^*^ | 393 | Glu | 1.65% | 1.47 | 568 | 2.38% |
| GAG | 175 | Glu | 0.73% | 0.53 |
| UGU^*^ | 224 | Cys | 0.94% | 1.48 | 302 | 1.27% |
| UGC | 78 | Cys | 0.33% | 0.52 |
| UGG | 475 | Trp | 1.99% | 1 | 475 | 1.99% |
| CGU^*^ | 356 | Arg | 1.49% | 1.34 | 1,595 | 6.68% |
| CGC | 95 | Arg | 0.40% | 0.36 |
| CGA^*^ | 357 | Arg | 1.50% | 1.34 |
| CGG | 116 | Arg | 0.49% | 0.44 |
| AGA^*^ | 489 | Arg | 2.05% | 1.84 |
| AGG | 182 | Arg | 0.76% | 0.68 |
| GGU^*^ | 597 | Gly | 2.51% | 1.33 | 1,802 | 7.55% |
| GGC | 186 | Gly | 0.78% | 0.41 |
| GGA^*^ | 716 | Gly | 3.01% | 1.59 |
| GGG | 303 | Gly | 1.27% | 0.67 |
| UAA^*^ | 406 | Stop | 1.70% | 1.34 | 616 | 2.58% |
| UAG | 188 | Stop | 0.79% | 0.88 |
| UGA | 22 | Stop | 0.09% | 0.78 |

**Table S2** Summary of codon usage and amino acids patterns of *A. episcopale* cp genome.

Note: * means RSCU ＞ 1
