# Supplementary material for: Comparative analysis of complete chloroplast genome of ethnodrug Aconitum episcopale and insight into its phylogenetic relationships
Source: Sci Rep. 2022 Jun 8;12:9439. doi: 10.1038/s41598-022-13524-3 (PMC9178047; doi:10.1038/s41598-022-13524-3)
Supplement: Supplementary file 4 — Supplementary Information 4. [file 41598_2022_13524_MOESM4_ESM.docx]

| **SSR nr.** | **Repeat Motif** | **Size** | **Sart** | **End** |
| --- | --- | --- | --- | --- |
| 1 | (A)_11_ | 11 | 2,565 | 2,575 |
| 2 | (A)_10_ | 10 | 3,610 | 3,619 |
| 3 | (TA)_7_ | 14 | 4,721 | 4,734 |
| 4 | (TA)_6_ | 12 | 5,205 | 5,216 |
| 5 | (A)_10_ | 10 | 5,896 | 5,905 |
| 6 | (A)_10_ | 10 | 6,044 | 6,053 |
| 7 | (TAAA)_3_ | 12 | 6,766 | 6,777 |
| 8 | (T)_10_ | 10 | 8,450 | 8,459 |
| 9 | (T)_10_ | 10 | 9,213 | 9,222 |
| 10 | (AAAT)_3_ | 12 | 13,751 | 13,762 |
| 11 | (T)_14_ | 14 | 18,741 | 18,754 |
| 12 | (AT)_5_ | 10 | 20,109 | 20,118 |
| 13 | (T)_10_ | 10 | 26,437 | 26,446 |
| 14 | (A)_10_ | 10 | 27,602 | 27,611 |
| 15 | (ATATT)_3_ | 15 | 30,323 | 30,337 |
| 16 | (TA)_5_ | 10 | 30,343 | 30,352 |
| 17 | (A)_10_ | 10 | 30,869 | 30,878 |
| 18 | (T)_10_ | 10 | 31,090 | 31,099 |
| 19 | (T)_12_ | 12 | 31,101 | 31,112 |
| 20 | (CTTT)_3_ | 12 | 31,244 | 31,255 |
| 21 | (AT)_5_ | 10 | 31,500 | 31,509 |
| 22 | (TA)_5_ | 10 | 32,716 | 32,725 |
| 23 | (A)_11_ | 11 | 33,001 | 33,011 |
| 24 | (AT)_5_ | 10 | 33,265 | 33,274 |
| 25 | (TA)_5_ | 10 | 34,035 | 34,044 |
| 26 | (A)_10_ | 10 | 34,750 | 34,759 |
| 27 | (AT)_9_ | 18 | 37,703 | 37,720 |
| 28 | (A)_11_ | 11 | 37,780 | 37,790 |
| 29 | (ATA)_4_ | 12 | 38,249 | 38,260 |
| 30 | (TA)_5_ | 10 | 48,545 | 48,554 |
| 31 | (A)_13_ | 13 | 48,764 | 48,776 |
| 32 | (T)_11_ | 11 | 50,217 | 50,227 |
| 33 | (A)_10_ | 10 | 50,904 | 50,913 |
| 34 | (AT)_5_ | 10 | 50,964 | 50,973 |
| 35 | (ATG)_4_ | 12 | 53,976 | 53,987 |
| 36 | (T)_10_ | 10 | 62,436 | 62,445 |
| 37 | (T)_10_ | 10 | 65,020 | 65,029 |
| 38 | (A)_11_ | 11 | 66,746 | 66,756 |
| 39 | (TA)_5_ | 10 | 67,907 | 67,916 |
| 40 | (TATT)_3_ | 12 | 68,273 | 68,284 |
| 41 | (A)_10_ | 10 | 69,007 | 69,016 |
| 42 | (TTCT)_3_ | 12 | 69,173 | 69,184 |
| 43 | (T)_16_ | 16 | 71,763 | 71,778 |
| 44 | (A)_10_ | 10 | 72,453 | 72,462 |
| 45 | (T)_10_ | 10 | 72,469 | 72,478 |
| 46 | (A)_11_ | 11 | 79,910 | 79,920 |
| 47 | (T)_10_ | 10 | 82,089 | 82,098 |
| 48 | (TAT)_5_ | 15 | 83,140 | 83,154 |
| 49 | (A)_11_ | 11 | 83,988 | 83,998 |
| 50 | (TTTC)_3_ | 12 | 84,518 | 84,529 |
| 51 | (T)_11_ | 11 | 84,849 | 84,859 |
| 52 | (A)_10_ | 10 | 94,748 | 94,757 |
| 53 | (TA)_7_ | 14 | 96,341 | 96,354 |
| 54 | (AATAA)_3_ | 15 | 112,670 | 112,684 |
| 55 | (TA)_5_ | 10 | 112,796 | 112,805 |
| 56 | (T)_10_ | 10 | 117,211 | 117,220 |
| 57 | (AT)_9_ | 18 | 120,666 | 120,683 |
| 58 | (ATA)_4_ | 12 | 124,796 | 124,807 |
| 59 | (T)_10_ | 10 | 125,198 | 125,207 |
| 60 | (ATT)_4_ | 12 | 126,735 | 126,746 |
| 61 | (ATA)_4_ | 12 | 126,768 | 126,779 |
| 62 | (CATT)_3_ | 12 | 127,333 | 127,344 |
| 63 | (AT)_7_ | 14 | 145,925 | 145,938 |
| 64 | (T)_10_ | 10 | 147,523 | 147,532 |

**Table S3** SSR lengths and repeat motifs identified in *A. episcopale* cp genome.
