# Supplementary material for: Comparative analysis of complete chloroplast genome of ethnodrug Aconitum episcopale and insight into its phylogenetic relationships
Source: Sci Rep. 2022 Jun 8;12:9439. doi: 10.1038/s41598-022-13524-3 (PMC9178047; doi:10.1038/s41598-022-13524-3)
Supplement: Supplementary file 5 — Supplementary Information 5. [file 41598_2022_13524_MOESM5_ESM.doc]

| **S/No** | **Repeat size** | **Repeat type** | **Repeat Position 1** | **Repeat Location 1** | **Repeat Position 2** | **Repeat Location 2** | **E-value** |
| --- | --- | --- | --- | --- | --- | --- | --- |
| 1 | 107 | F | 70,177 | *rpl20* | 70,195 | *rpl20* | 8.33E-53 |
| 2 | 89 | F | 70,177 | *rpl20* | 70,213 | *rpl20* | 4.76E-42 |
| 3 | 71 | F | 70,177 | *rpl20* | 70,231 | *rpl20* | 2.61E-31 |
| 4 | 72 | P | 65,115 | IGS | 65,115 | IGS | 7.04E-30 |
| 5 | 53 | F | 70,177 | *rpl20* | 70,249 | *rpl20* | 1.34E-20 |
| 6 | 52 | F | 40,732 | *psaB* | 42,956 | *psaA* | 2.01E-16 |
| 7 | 42 | F | 95,233 | *ycf15* | 147,004 | *ycf15* | 3.53E-16 |
| 8 | 42 | P | 95,233 | *ycf15* | 95,233 | *ycf15* | 3.53E-16 |
| 9 | 42 | P | 147,004 | *ycf15* | 147,004 | *ycf15* | 3.53E-16 |
| 10 | 48 | P | 79,762 | IGS | 79,762 | IGS | 8.75E-16 |
| 11 | 39 | F | 45,713 | IGS | 100,674 | IGS | 2.26E-14 |
| 12 | 39 | P | 45,713 | IGS | 141,566 | IGS | 2.26E-14 |
| 13 | 35 | F | 70,177 | *rpl20* | 70,267 | *rpl20* | 6.07E-10 |
| 14 | 31 | F | 6,382 | IGS | 6,413 | *IGS* | 1.48E-09 |
| 15 | 30 | P | 8,141 | IGS | 47,443 | *trnS-GGA* | 5.92E-09 |
| 16 | 36 | R | 70,176 | *rpl20* | 70,251 | *rpl20* | 2.79E-07 |
| 17 | 36 | R | 70,176 | *rpl20* | 70,233 | *rpl20* | 2.79E-07 |
| 18 | 36 | R | 70,176 | *rpl20* | 70,215 | *rpl20* | 2.79E-07 |
| 19 | 36 | R | 70,176 | *rpl20* | 70,197 | *rpl20* | 2.79E-07 |
| 20 | 36 | R | 70,176 | *rpl20* | 70,179 | *rpl20* | 2.79E-07 |
| 21 | 33 | R | 70,197 | *rpl20* | 70,251 | *rpl20* | 4.40E-07 |
| 22 | 33 | R | 70,197 | *rpl20* | 70,233 | *rpl20* | 4.40E-07 |
| 23 | 33 | R | 70,197 | *rpl20* | 70,215 | *rpl20* | 4.40E-07 |
| 24 | 33 | R | 70,197 | *rpl20* | 70,197 | *rpl20* | 4.40E-07 |
| 25 | 33 | R | 70,215 | *rpl20* | 70,251 | *rpl20* | 4.40E-07 |
| 26 | 33 | R | 70,215 | *rpl20* | 70,233 | *rpl20* | 4.40E-07 |
| 27 | 33 | R | 70,215 | *rpl20* | 70,215 | *rpl20* | 4.40E-07 |
| 28 | 33 | R | 70,233 | *rpl20* | 70,251 | *rpl20* | 4.40E-07 |
| 29 | 33 | R | 70,233 | *rpl20* | 70,233 | *rpl20* | 4.40E-07 |
| 30 | 33 | R | 70,251 | *rpl20* | 70,251 | *rpl20* | 4.40E-07 |
| 31 | 32 | P | 4,316 | IGS | 4,316 | IGS | 1.65E-06 |
| 32 | 34 | R | 70,178 | *rpl20* | 70,269 | *rpl20* | 3.74E-06 |
| 33 | 34 | R | 70,196 | *rpl20* | 70,269 | *rpl20* | 3.74E-06 |
| 34 | 34 | R | 70,214 | *rpl20* | 70,269 | *rpl20* | 3.74E-06 |
| 35 | 34 | R | 70,232 | *rpl20* | 70,269 | *rpl20* | 3.74E-06 |
| 36 | 34 | R | 70,250 | *rpl20* | 70,269 | *rpl20* | 3.74E-06 |
| 37 | 33 | F | 8,138 | IGS | 37,506 | IGS | 1.36E-05 |
| 38 | 31 | F | 93,198 | *ycf2* | 93,216 | *ycf2* | 1.80E-04 |
| 39 | 31 | P | 93,166 | *ycf2* | 149,032 | *ycf2* | 1.80E-04 |
| 40 | 31 | P | 93,184 | *ycf2* | 149,050 | *ycf2* | 1.80E-04 |
| 41 | 31 | F | 149,004 | *ycf2* | 149,050 | *ycf2* | 1.80E-04 |
| 42 | 30 | P | 37,510 | IGS | 47,443 | *trnS-GGA* | 6.49E-04 |
| 43 | 30 | P | 72,433 | IGS | 72,452 | IGS | 6.49E-04 |
| 44 | 30 | P | 76,227 | IGS | 76,,275 | IGS | 6.49E-04 |

**Table S4.** The distribution of long repeats sequences in *A. episcopale* chloroplast genome.
