# Supplementary material for: Comparative analysis of complete chloroplast genome of ethnodrug Aconitum episcopale and insight into its phylogenetic relationships
Source: Sci Rep. 2022 Jun 8;12:9439. doi: 10.1038/s41598-022-13524-3 (PMC9178047; doi:10.1038/s41598-022-13524-3)
Supplement: Supplementary file 6 — Supplementary Information 6. [file 41598_2022_13524_MOESM6_ESM.doc]

| **No.** | **Consensus size × copy number** | **Start** | **End** | **Repeat length (bp)** | **Location** |
| --- | --- | --- | --- | --- | --- |
| 1 | 11×2.6 | 4,728 | 4,757 | 30 | *trnK-UUU/rps16*(IGS) |
| 2 | 23×2.0 | 5,800 | 5,844 | 45 | *rsp16/trnQ-UUG*(IGS) |
| 3 | 31×2.0 | 6,383 | 6,444 | 62 | *rsp16/trnQ-UUG*(IGS) |
| 4 | 19×2.0 | 8,258 | 8,295 | 38 | *trnS-GCU/trnG-GCC*(IGS) |
| 5 | 20×1.9 | 13,364 | 13,401 | 38 | *atpF/ atpH*(IGS) |
| 6 | 21×2.0 | 27,149 | 27,190 | 42 | *rpoB/ trnC-GCA*(IGS) |
| 7 | 17×2.1 | 44,497 | 44,530 | 34 | *petN/psbM*(IGS) |
| 8 | 24×2.0 | 48,422 | 48,467 | 46 | *psaA/ ycf3*(IGS) |
| 9 | 18×2.0 | 48,688 | 48,723 | 36 | *rps4/trnT-UGU*(IGS) |
| 10 | 23×2.1 | 48,953 | 49,000 | 48 | *trnT-UGU/trnL-UAA*(IGS) |
| 11 | 18×6.9 | 70,178 | 70,302 | 125 | *rpl20*(CDS) |
| 12 | 13×2.0 | 73,180 | 73,205 | 26 | *clpP*(intron) |
| 13 | 24×2.0 | 81,383 | 81,430 | 48 | *rps11*(CDS) |
| 14 | 21×2.0 | 85,614 | 85,655 | 42 | *rpl22*(CDS) |
| 15 | 22×3.2 | 90,760 | 90,828 | 69 | *ycf2*(CDS) |
| 16 | 15×2.7 | 93,172 | 93,212 | 41 | *ycf2*(CDS) |
| 17 | 18×2.6 | 93,201 | 93,247 | 47 | *ycf2*(CDS) |
| 18 | 21×2.8 | 101,037 | 101,091 | 55 | *rps12/trnV-GAC*(IGS) |
| 19 | 2×21.5 | 120,641 | 120,683 | 43 | *ndhG/ndhI* |
| 20 | 24×2.0 | 126,425 | 126,472 | 48 | *ycf1*(CDS) |
| 21 | 24×2.3 | 127,602 | 127,655 | 54 | *ycf1(*CDS) |
| 22 | 12×2.1 | 128,837 | 128,861 | 25 | *ycf1*(CDS) |
| 23 | 20×2.8 | 141,189 | 141,243 | 55 | *trnV-GAC/rps12*(IGS) |
| 24 | 18×2.6 | 149,033 | 149,079 | 47 | *ycf2*(CDS) |
| 25 | 15×2.7 | 149,068 | 149,108 | 41 | *ycf2*(CDS) |
| 26 | 21×3.2 | 151,453 | 151,520 | 68 | *ycf2*(CDS) |

**Table S5.** Distribution of tandem repeats in the *A. episcopale* chloroplast genome. IGS: Intergenic spacer; CDS: protein-coding region.
